# Bias Evaluation in Medical Applications of Large Language Models: A Systematic Review

**DOI:** 10.1192/bjo.2026.11056

**Published:** 2026-06-30

**Authors:** Priya Gupta, Shweta Girish Rao, Rukhma Mubarika, Shweta Velmurugan, Judith Harrison

**Affiliations:** 1 School of Medicine, Newcastle University, Newcastle upon Tyne, United Kingdom; 2 School of Medicine, University of Sunderland, Sunderland, United Kingdom; 3 Translational and Clinical Research Institute, and ^3^rd Floor, Biomedical Research Building Campus for Ageing and Vitality, Newcastle upon Tyne, United Kingdom

## Abstract

**Aims:**

Large language models (LLMs) are increasingly evaluated and deployed in healthcare, yet unrecognised bias risks reinforcing inequities. This risk is particularly salient in psychiatry, where biased outputs could influence risk formulation, diagnostic reasoning, triage, and therapeutic communication. We examined whether and how bias is evaluated in studies of medical applications of LLMs, and summarised methodologies, reporting practices,and limitations of current bias detection and mitigation approaches to support safe and trustworthy implementation.

**Methods:**

We searched databases (EMBASE, MEDLINE, PsycINFO, PubMed, ACL Anthology, ACM Digital Library, arXiv, medRxiv, and bioRxiv; 2017–June 2025), following PRISMA guidance. After de-duplication, >1,800 records were retrieved for title/abstract screening. We assessed 3,664 full-text articles against pre-specified eligibility criteria. Studies were included if they evaluated a medical application of an LLM and reported any explicit bias assessment. Studies that otherwise met eligibility for medical LLM evaluation but did not assess bias were recorded separately to quantify the evidence gap. Screening and data extraction were conducted by multiple reviewers with disagreements resolved by discussion. We extracted specialty (including psychiatry), use case (e.g. decision support, documentation, patient communication), evaluation setting (benchmark/vignette vs clinical data), protected attributes and bias targets assessed, bias methods/metrics, and mitigation strategies proposed or tested. Findings were synthesised narratively with descriptive counts across specialties and use cases.

**Results:**

Of 3,664 full texts screened, 278 studies met inclusion by explicitly assessing bias. A further 757 studies evaluated medical applications of LLMs and otherwise met eligibility criteria but did not report any bias assessment, indicating that most medical LLM evaluations omit bias evaluation. Included studies spanned clinical decision support, medical documentation, patient communication, education, and biomedical research across multiple specialties. Bias assessment rates varied by specialty: psychiatry 21/180 (11.7%), radiology 7/107 (6.5%), and oncology 5/51 (9.8%). Bias assessments most commonly examined demographic bias (gender, race/ethnicity, age) and variation in symptom or diseasepresentation. Methodologies included prompt-based probing (e.g. clinical vignettes), counterfactual testing, and red teaming to elicit biased responses; statistical comparisons of performance across groups (including fairness-adjusted performance metrics such as the FAP score); and clinician-led safety evaluation frameworks assessing output suitability. Proposed mitigation strategies included more diverse training data, reinforcement learning with human feedback (RLHF), direct preference optimisation, and ongoing human oversight.

**Conclusion:**

Despite rapid growth in medical LLM research, explicit bias assessment remains inconsistent and frequently absent. Standardised, transparent bias evaluation across patient populations, tasks, and clinical contexts is needed to support safe implementation and ensure equitable provision.

